# Aging in Rats Differentially Affects Markers of Transcriptional and Translational Capacity in Soleus and Plantaris Muscle

**DOI:** 10.3389/fphys.2017.00518

**Published:** 2017-07-20

**Authors:** Christopher B. Mobley, Petey W. Mumford, Wesley C. Kephart, Cody T. Haun, Angelia M. Holland, Darren T. Beck, Jeffrey S. Martin, Kaelin C. Young, Richard G. Anderson, Romil K. Patel, Gillis L. Langston, Ryan P. Lowery, Jacob M. Wilson, Michael D. Roberts

**Affiliations:** ^1^School of Kinesiology, Auburn University Auburn, AL, United States; ^2^Edward via College of Osteopathic Medicine Auburn, AL, United States; ^3^Applied Science and Performance Institute Tampa, FL, United States; ^4^Department of Health and Human Performance, Concordia University Chicago River Forest, IL, United States

**Keywords:** fast-twitch muscle, aging, atrophy, muscle protein synthesis, mRNA, ribosome

## Abstract

Alterations in transcriptional and translational mechanisms occur during skeletal muscle aging and such changes may contribute to age-related atrophy. Herein, we examined markers related to global transcriptional output (i.e., myonuclear number, total mRNA and RNA pol II levels), translational efficiency [i.e., eukaryotic initiation and elongation factor levels and muscle protein synthesis (MPS) levels] and translational capacity (ribosome density) in the slow-twitch soleus and fast-twitch plantaris muscles of male Fischer 344 rats aged 3, 6, 12, 18, and 24 months (*n* = 9–10 per group). We also examined alterations in markers of proteolysis and oxidative stress in these muscles (i.e., 20S proteasome activity, poly-ubiquinated protein levels and 4-HNE levels). Notable plantaris muscle observations included: (a) fiber cross sectional area (CSA) was 59% (*p* < 0.05) and 48% (*p* < 0.05) greater in 12 month vs. 3 month and 24 month rats, respectively, suggesting a peak lifetime value near 12 months and age-related atrophy by 24 months, (b) MPS levels were greatest in 18 month rats (*p* < 0.05) despite the onset of atrophy, (c) while regulators of ribosome biogenesis [c-Myc and upstream binding factor (UBF) protein levels] generally increased with age, ribosome density linearly decreased from 3 months of age and RNA polymerase (Pol) I protein levels were lowest in 24 month rats, and d) 20S proteasome activity was robustly up-regulated in 6 and 24 month rats (*p* < 0.05). Notable soleus muscle observations included: (a) fiber CSA was greatest in 6 month rats and was maintained in older age groups, and (b) 20S proteasome activity was modestly but significantly greater in 24 month vs. 3/12/18 month rats (*p* < 0.05), and (c) total mRNA levels (suggestive of transcriptional output) trended downward in older rats despite non-significant between-group differences in myonuclear number and/or RNA Pol II protein levels. Collectively, these findings suggest that plantaris, not soleus, atrophy occurs following 12 months of age in male Fisher rats and this may be due to translational deficits (i.e., changes in MPS and ribosome density) and/or increases in proteolysis rather than increased oxidative stress and/or alterations in global transcriptional mechanisms.

## Introduction

Aging in humans is associated with declines in muscle mass and function (Doherty, 2003). In this regard, a landmark study by Lexell et al. (1988) reported that *vastus lateralis* muscle fiber cross-sectional area (CSA) was greatest in males 25 years of age, values of which were ~90% higher than those observed in 80 year old males. Moreover, these authors reported a 1% per year decrease in muscle fiber CSA beginning after 25 years of age (Lexell et al., 1988; Nilwik et al., 2013), and more recent papers have reported similar findings (Goodpaster et al., 2006). Researchers have attributed age-related decreases in muscle fiber size to a variety of mechanistic factors including, but not limited to, a reduction in basal muscle protein synthesis (MPS) rates (Balagopal et al., 1997), a decrease in the anabolic response to meal feeding (Cuthbertson et al., 2005), a decrease in mitochondrial quality with aging (Murgia et al., 2017), an increase in low-grade inflammation (Schaap et al., 2006) and a decrease in the quantity and function of satellite cells (Kadi et al., 2004). Losses in spinal motor neurons and motor units also become apparent by the sixth decade in life (Piasecki et al., 2016), and motor unit behavior and morphology (e.g., conduction velocity, remodeling of the neuromuscular junction, axonal atrophy, and axonal filament accretion) become evident with aging (Manini et al., 2013). Skeletal muscle fibroblast content also increases with aging which leads to an increase in extracellular matrix collagen content and an increase in advanced glycation end-product cross-links (Kragstrup et al., 2011), and several reports suggest that skeletal muscle markers of oxidative stress (e.g., mitochondrial DNA damage and protein/lipid oxidation) increase with aging which may result in functional decrements of metabolic/mitochondrial enzymes and/or contractile protein apparatuses (Aoi and Sakuma, 2011). Hence, the aging milieu of skeletal muscle is complex and involves many of the aforementioned factors, and it is apparent that functional decrements in muscle strength (Kerksick et al., 2016) and oxidative capacity (Nair, 2005) occur as a result.

Ribosome biogenesis involves the formation of new ribosomes, which are the organelles that catalyze MPS, and the rate-limiting step of ribosome biogenesis involves the coordinated action of various transcription factors [e.g., c-Myc, upstream binding factor (UBF) and others] recruiting RNA polymerase I (Pol I) to the promoter region of rDNA in order to facilitate 47S pre-rRNA transcription (Chaillou et al., 2014). It has been suggested that the maintenance of ribosome function through the coordinated action of various eukaryotic initiation and elongation factors as well as translational capacity through ribosome biogenesis is necessary for muscle mass maintenance (Kimball et al., 1992; Chaillou et al., 2014; Wen et al., 2016). Furthermore, aging is also associated with decrements in eukaryotic initiation factors (Kimball et al., 1992) as well as ribosome biogenesis (Chaillou, 2017), and both of these phenomena may contribute to age-related muscle atrophy. Aside from these variables being potentially compromised with aging, total mRNA levels (which is a surrogate of transcriptional capacity) have also been reported to be lower in certain tissues in aged rodents (De Cecco et al., 2013), and the authors from both studies suggest that reductions in global transcriptional output may result in cellular senescence. Therefore, the purpose of the current study was to investigate the effects of aging on biomarkers related to global transcription and translation in soleus (predominantly slow-twitch) and plantaris (predominantly fast-twitch) muscles over the lifespan of Fischer 344 male rats aged 3, 6, 12, 18, and 24 months old. We hypothesized that age-related atrophy of the plantaris muscle would occur whereas soleus muscle atrophy would not occur. We further hypothesized that age-related plantaris atrophy would coincide with the decrement in markers related to transcriptional output, translational efficiency and/or translational capacity. As a secondary aim, we sought to examine how plantaris and soleus muscle markers related to oxidative stress and proteolysis were affected over the life span in these rodents.

## Materials and methods

### Animal experimental procedures

All procedures involving animal husbandry and experimentation were approved by Auburn University's Institutional Animal Care and Use Committee (protocol #2015-2790). Male, Fischer 344 rats (300–600 g) aged 3, 6, 12, 18, and 24 months (*n* = 9–10 rats per age group) were purchased from Envigo Laboratories (Indianapolis, IN, USA), and housed two per cage on week prior to experimentation. During this time, animal quarters were maintained on a constant 12 h light: 12 h dark cycle at ambient room temperature, and tap water and standard rodent chow (24% protein, 58% carbohydrate, 18% fat; Teklad Global #2018 Diet, Envigo Laboratories) were provided to animals *ad libitum*.

The day prior to experimentation, rotarod performance was assessed using a single-lane device (Product#: ENV-571R; Med Associates Inc., Saint Albans City, VT, USA). All assessments took place during the beginning of the rat light cycle (i.e., 0600–0800) whereby rats were placed on the device and the motorized rotor was initiated at a progressive speed from 4.0 to 40.0 revolutions/min. An automated timer tracked time spent on the rod and, once the rats fatigued and dismounted from the rod, a laser beam break stopped the timer. Notably, rotarod performance is used in rodent studies to assess a combination of balance, grip strength, motor coordination and muscular endurance (Hamm et al., 1994).

The morning of experimentation, animals were removed from their quarters between 0600 and 0700, transported to the Molecular and Applied Sciences Laboratory in the School of Kinesiology building and immediately tested for forelimb strength using a digital strength meter (Product#: BIO-G53; Bioseb, Vitrolles, FRA). Briefly, forelimb strength testing involved placing the animals' two forelimb paws on a metal grid adjoined to an electronic force transducer. The animal was then horizontally pulled away from the grid and force readings were provided upon the point at which the animal could no longer maintain grip contact. This test was repeated three times per the recommendations of Deacon (2013), and the average of all three trails was used for statistical analyses.

Following the strength testing, animals were allowed to acclimate for 3–4 h with water but without food access. 30 min prior to euthanasia, animals were administered puromycin dihydrochloride (Ameresco; Solon, OH, USA) (0.021 mg/kg in 1 mL of diluted in phosphate buffered saline, intraperitoneal injection) in order to determine relative MPS levels via the Surface Sensing of Translation (SUnSET) method (Goodman and Hornberger, 2013). Rats were then euthanized under CO_2_ gas induction in a 2 L chamber (VetEquip, Inc., Pleasanton, CA, USA). Following euthanasia, body masses were recorded, right-leg plantaris and soleus muscles were dissected out, and muscles were weighed using an analytical scale with a sensitivity of 0.0001 g (Mettler-Toledo; Columbus, OH, USA). During dissection muscles were cut in very close proximity at the origin and insertion sites and visible connective tissue at the insertion site was removed. Muscles were then processed for biochemical assays the day of euthanasia as described in the following paragraphs.

### Tissue preparation on the day of euthanasia

For protein analyses, ~50 mg of plantaris and soleus muscles from each rat were placed in 1.7 mL microcentrifuge tubes containing 500 μL of ice-cold cell lysis buffer [20 mM Tris-HCl (pH 7.5), 150 mM NaCl, 1 mM Na_2_EDTA, 1 mM EGTA, 1% Triton, 2.5 mM sodium pyrophosphate, 1 mM β-glycerophosphate, 1 mM Na_3_VO_4_, 1 μg/mL leupeptin; Cell Signaling, Danvers, MA, USA] pre-stocked with protease and Tyr/Ser/Thr phosphatase inhibitors. Samples were then homogenized via micropestle manipulation, insoluble proteins were removed with centrifugation at 500 g for 5 min, and supernatants were stored at −80°C prior to Western blotting described below. For histological analyses, ~50 mg of plantaris and soleus muscle obtained from the mid-belly of the muscle were embedded in cryomolds containing freezing media (Tissue-Tek®, Sakura Finetek Inc.; Torrence, CA, USA) per the methods of Bennett et al. (2013). Cryomolds were frozen using liquid nitrogen-cooled isopentane and were then stored at −80°C until immunofluorescent staining for fiber cross sectional area (CSA) and myonuclear number described below. Specifically, embedding was performed per the directions of Kumar et al. (2015) whereby tissue was laid in the cryomolds in the correct orientation for perpendicular slicing in a non-stretched state prior to rapid freezing. For total RNA, total mRNA and real-time polymerase chain reaction (RT-PCR) analyses, ~30 mg of plantaris and soleus muscles from each rat were homogenized in 1.7 mL microcentrifuge tubes containing 500 μL of Ribozol (Ameresco; Solon, OH, USA) via micropestle manipulation and RNA isolation was performed per manufacturer recommendations. Thereafter, samples were frozen at −80°C until RNA quantification, mRNA isolation and cDNA synthesis described in greater detail below. Notably, total RNA was used as a surrogate for ribosome density as in past publications (Nader et al., 2005; Mobley et al., 2016a,b) given that 85+% of total RNA is ribosomal RNA (rRNA) and two-thirds of the ribosome is made up of rRNA. Hence, changes in total RNA were presumed to represent changes in ribosome density herein.

### Western blotting

Cell lysates obtained through cell lysis buffer processing (described above) were batch process-assayed for total protein content using a BCA Protein Assay Kit (Thermo Fisher Scientific; Waltham, MA, USA). Thereafter, homogenates were prepared for Western blotting using 4x Laemmli buffer at 2 μg/μL. MPS determination via the Western blotting SUnSET method were performed as previously detailed by our laboratory (Mobley et al., 2016a). Membrane development was performed using an enhanced chemiluminescent reagent (Amersham, Pittsburgh, PA, USA), and band densitometry was performed through the use of a digitized gel documentation system and associated densitometry software (UVP; Upland, CA, USA). Specifically, whole-lane densitometry using rectangular regions of interest was performed to determine relative MPS levels, and these values were divided by respective Ponceau lane densities to control for potential variations in protein loading. Similar Western blotting methods were also used to assess plantaris and soleus protein levels of poly-ubiquinated proteins (poly-Ub; primary antibody from Cell Signaling), RNA Pol I (Thermo Fisher Scientific), RNA Pol II, c-Myc (Cell Signaling), UBF (Santa Cruz, Dallas, TX, USA), eukaryotic initiation factor (eIF) 4E (Cell Signaling), eIF2Bε (Cell Signaling), eukaryotic elongation factor (eEF) 2 (Cell Signaling), and 4-hydroxynonenal- conjugated proteins (4-HNE; Abcam, Cambridge, MA, USA). Notably, all primary antibody dilutions were 1:1,000 in Tris buffered saline +0.1% Tween 20 (TBST) and all secondary antibody dilutions were 1:2,000 in TBST.

### 20S proteasome activity assay

20S proteasome activity assays on plantaris and soleus cell lysates were performed using a commercially available fluorometric kit per the manufacturer's instructions (EMD Millipore, Billerica, MA, USA).

### Immunohistochemistry

Immunohistocheonstry for fiber typing and fiber CSA assessments were performed similar to the methods previously performed by our laboratory (Hyatt et al., 2015; Mobley et al., 2016b). Briefly, 20 μm frozen sections were cut from the cryomolds mentioned above using a cryostat (Leica Biosystems; Buffalo Grove, IL, USA), and placed on charged microscope slides (Azer Scientific, Morgantown, PA).

For non-fiber type staining (sections used for fiber CSA, myonuclear number and myonuclear domain analyses), slides were processed similarly as described above with the exception being that the primary antibody solution contained only rabbit anti-dystrophin IgG (Thermo Fisher Scientific) and the secondary antibody solution contained Texas Red-conjugated anti-rabbit IgG (Vector Laboratories). Following secondary antibody incubations, slides were washed for 5 min in 1x PBS, air-dried and were mounted with a solution containing 4,6-Diamidino-2-phenylindole, dihydrochloride (DAPI) (Vector Laboratories) for nuclear staining. Following mounting, slides were stored in the dark at 4°C until immunofluorescent images were obtained. Images were obtained using a 20x objective via a fluorescent microscope as described above and muscle fiber CSA, myonuclear number and myonuclear domain were manually quantified from at least 50 fibers per muscle using scaling and counting tools provided by open-sourced software (imageJ; National Institutes of Health, Bethesda, MD, USA).

For fiber type staining, slides were incubated in 1x phosphate-buffered saline (PBS) with 0.5% Triton X-100 (Ameresco) for 5 min. Slides were then washed for 5 min in 1x PBS and incubated for 60 min with a primary antibody solution containing rabbit anti-dystrophin IgG (Thermo Fisher Scientific; 10 μL antibody per 1 mL of blocking solution), mouse anti-myosin type I IgM (catalog #: A4.840; Hybridoma Bank, Iowa City, IA, USA; 100 μL per 1 mL of blocking solution), and mouse anti-myosin IIa IgG (catalog #: SC71; Hybridoma Bank; 100 μL per 1 mL of blocking solution). Slides were then washed for 5 min in 1x PBS and incubated in the dark for 60 min with a secondary antibody solution containing Texas Red-conjugated anti-rabbit IgG (Vector Laboratories, Burlingame, CA), Alexa Fluor 350 (Thermo Fisher Scientific) and Alexa Fluor 488 (Thermo Fisher Scientific) (10 μL of all secondary antibodies per 1 mL of blocking solution). Slides were then washed for 5 min in 1x PBS, air-dried and were mounted with fluorescent mounting media (Vector Laboratories). Following mounting, slides were stored in the dark at 4°C until immunofluorescent images were obtained. Images were obtained using a 20x objective via a fluorescent microscope (Nikon Eclipse Ti-U; Nikon Inc., Tokyo, Japan) and associated software. Fiber type counting was performed manually as previously published by our laboratory and others (McClung et al., 2007; Hyatt et al., 2015) whereby blue cell bodies (detected by the DAPI filter) were counted as type I fibers, green cell bodies (detected by the FITC filter) as type IIa fibers, and black cell bodies (unlabeled) as type IIx fibers. Notably, *n* = 5–8 animals per age group were fiber type assayed for each muscle due to limited sections following non fiber type staining.

### RNA isolation and real-time PCR

Total RNA concentrations from isolated RNA described above were determined in duplicate using a NanoDrop Lite spectrophotometer (Thermo Fisher Scientific). One microgram of plantaris and soleus muscle RNA was reverse transcribed into cDNA for RT-PCR analysis with cDNA synthesis reagents (Quanta Biosciences, Gaithersburg, MD) per the manufacturer's recommendations. Real-time PCR was performed using gene-specific primers and SYBR green chemistry (Quanta Biosciences). Primer sequences used were as follows: pre-45S rRNA forward primer 5′-TGGGGCAGCTTTATGACAAC3′ reverse primer 5′TAGCACCAAACGGGAAAACC3′; rpS16 (housekeeping gene for plantaris): forward primer 5′- TCGCTGCGAATCCAAGAAGT-3′ reverse primer 5′- CCCTGATCCTTGAGACTGGC-3′; HDAC1 (housekeeping gene for soleus): forward primer 5′-GAGCGGTGATGAGGATGAGG-3′ reverse primer 5′-CACAGGCAATGCGTTTGTCA-3′. Pre-45S rRNA fold-change values relative to 3 month rats were performed using the Livak method (i.e., 2^−ΔΔCT^ assuming 100% primer binding efficiency), where: a) 2^−ΔCT^ = (housekeeping gene) CT − gene of interest CT, and b) 2^−ΔΔCT^ (or fold-change) = [2^−ΔCT^ value of 6/12/18/24 month rat/2^−ΔCT^ average of 3 month rat group]. Of note, the housekeeping genes used for each muscle remained stable across all treatments, and melt curve analyses were performed during each PCR reaction to confirm that only one PCR product was obtained.

### mRNA isolation

Messenger RNA (mRNA) was isolated from plantaris and soleus total RNA pellets using a magnetic poly A+ RNA isolation kit (New England BioLabs Inc., Ipswich, MA, USA). Samples were processed according to manufacturers' instructions. Following isolation, mRNA concentration was determined in duplicate using the NanoDrop Lite spectrophotometer (Thermo Fisher Scientific) and coefficient of variation values for duplicate readings were 15.2%.

### Statistics

All data are presented in figures as means ± standard error of the mean (SE) values. Statistics were performed using SPSS v22.0 (IBM, Armonk, NY, USA). All dependent variables were compared between age groups using one-way ANOVAs with protected LSD *post-hoc* tests being performed if ANOVA *p* < 0.05. Select associations were also performed using bivariate correlations (described in Results), and correlations were considered significant at *p* < 0.05.

## Results

### Body mass, muscle mass, forelimb strength, and rotarod differences between age groups

**Figure 1:**
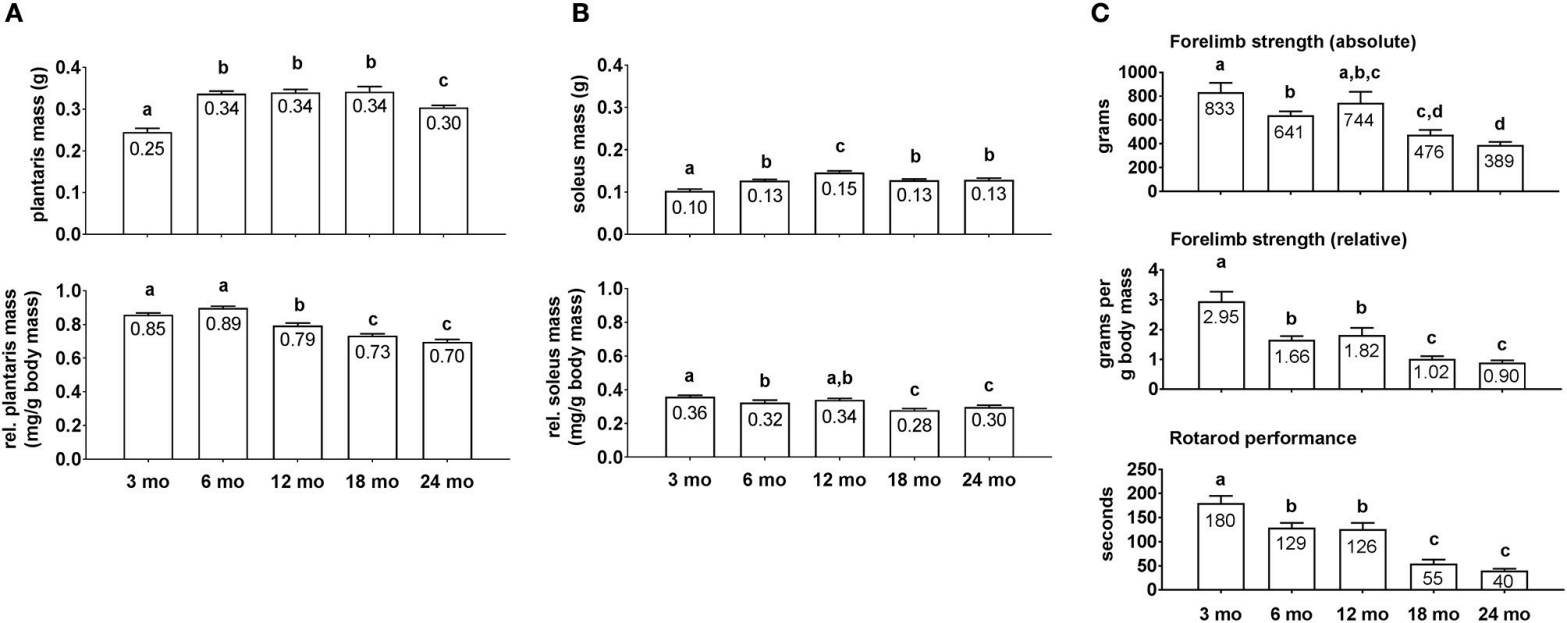
Plantaris mass, soleus mass, forelimb strength, and rotarod differences between age groups. Age group differences in raw plantaris mass (non-body mass-adjusted) (**A**, upper inset), relative plantaris mass (body mass-adjusted) (**A**, lower inset), raw soleus mass (**B**, upper inset), relative soleus mass (**B**, lower inset), absolute forelimb strength (**C**, upper inset), relative forelimb strength (**C**, middle inset) and rotarod performance (**C**, lower inset). Data are presented as mean + SE and mean values for each dependent variable are presented within each bar. Different superscript letters indicate significant between-group differences indicated by one-way ANOVAs and protected LSD *post-hoc* tests (*p* < 0.05). mo, month.

Average group body masses at the time of sacrifice were as follows: 3 month = 285 ± 10 g, 6 month = 399 ± 24 g, 12 month = 429 ± 7 g, 18 month = 466 ± 14 g, and 24 month = 439 ± 13 g (*n* = 9–10 rats per age group). Notably, 3 month rats weighed less than all other age groups (*p* < 0.05), 6 month rats weighed less than 18 month and 24 month rats (*p* < 0.05), and there were no differences in body masses between the 12/18/24 month groups. Raw plantaris mass (Figure 1A, upper inset) was ~35% greater in 6/12/18 month rats compared to 3 month rats (*p* < 0.05), and was 13% lower in 24 month vs. 18 month rats (*p* < 0.05). Relative plantaris mass (Figure 1A, low inset) was not statistically different between 3 and 6 month rats, 13% lower in 12 month vs. 6 month rats (*p* < 0.05), was 8% lower in 18 month vs. 12 month rats (*p* < 0.05), and not statistically different between 18 and 24 month rats. Raw soleus mass (Figure 1B, upper inset) was 30% greater in 6 month vs. 3 month rats (*p* < 0.05), 15% greater in 12 month vs. 6 month rats (*p* < 0.05), 15% lower in 18 month vs. 12 month rats (*p* < 0.05), and not statistically different between 18 and 24 month rats. Relative soleus mass (Figure 1B, lower inset) was 13% higher in 3 month vs. 6 month rats (*p* < 0.05), not statistically different between 3 or 6 month vs. 12 month rats, 21% lower in 18 month vs. 12 month rats (*p* < 0.05), and not statistically different between 18 and 24 month rats. Collectively, these data suggest that peak raw plantaris and soleus mass values occur ~12 month of age, and relative masses peaked ~3–12 month of age.

Absolute forelimb grip strength values (Figure 1C, upper inset) displayed near linear decreases across age groups. Specifically, 3 month rats possessed 30% greater values compared to 6 month rats (*p* < 0.05), there was no significant difference between 3 and 12 month rats, and 24 month rats exhibited the lowest values which were ~50+% less than the 3/6/12 month groups (*p* < 0.05). Relative forelimb grip strength values (Figure 1C, middle inset) displayed near linear decreases across age groups as well. Specifically, 3 month rats possessed 62–78% greater values compared to 6 and 12 month rats (*p* < 0.05), and the 18/24 month groups possessed values that were 40+% less than the 6/12 month groups (*p* < 0.05). As with strength values, rotarod performance times also displayed near linear decreases across age groups (Figure 1C, lower inset). Specifically, 3 month rats possessed values that were ~40% greater than the 6/12 month groups, and the 18/24 month groups possessed values that were ~60% less than the 6/12 month groups (*p* < 0.05).

### Fiber type distributions in the soleus and plantaris muscles between age groups

**Figure 2:**
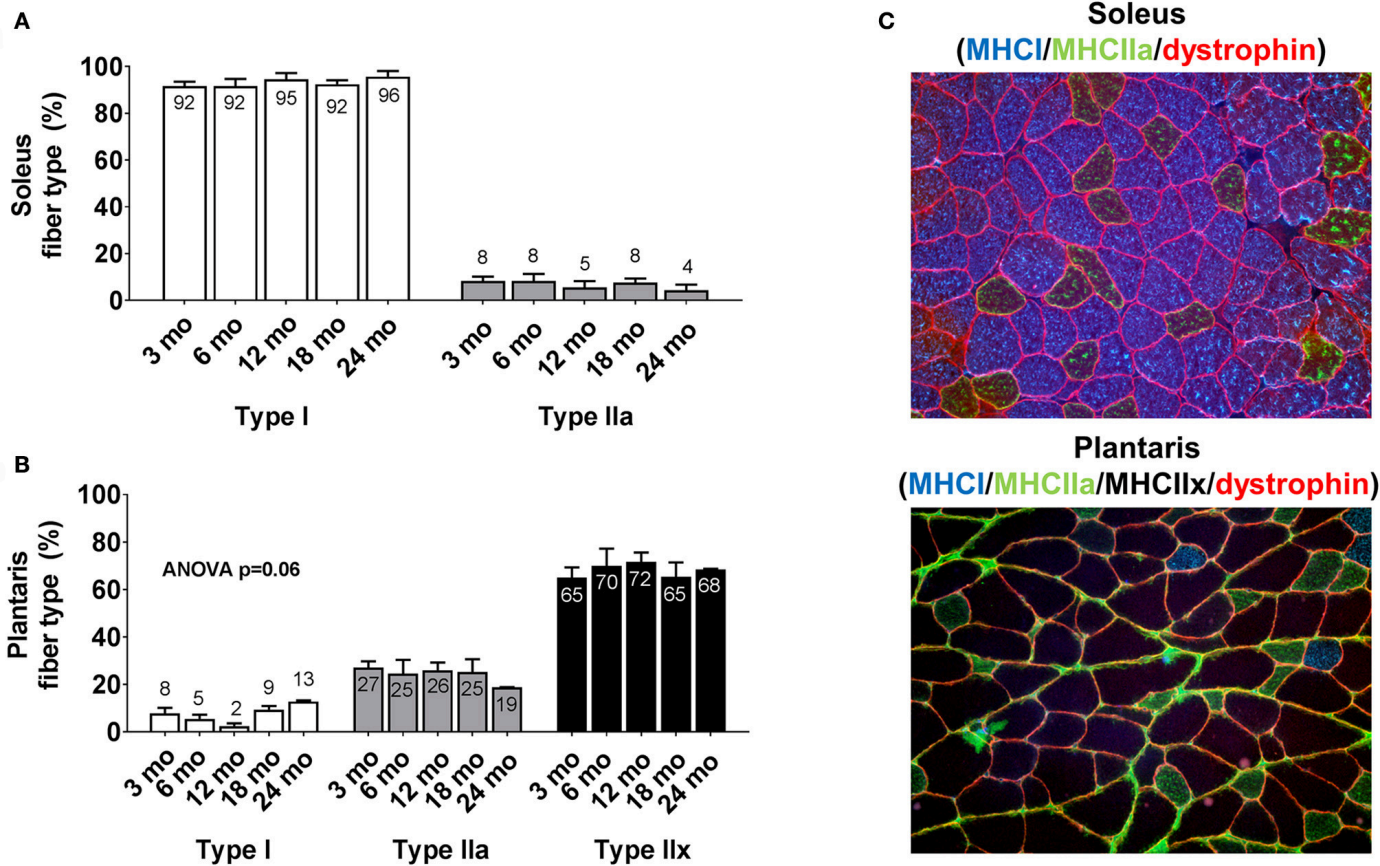
Plantaris and soleus fiber type distribution differences between age groups. Age group differences in soleus fiber type **(A)** and plantaris fiber type **(B)**. Data are presented as mean + SE and mean values for each dependent variable are presented within each bar. **(C)** Illustrates representative 20x objective micrographs of each stain. mo, month.

There were no significant between-group differences in soleus type I or type IIa fiber percentages between age groups (Figure 2A). Likewise, there were no significant between-group differences in plantaris type I, type IIa or type IIx fiber percentages between age groups (Figure 2B). The cumulative soleus fiber type percentage for all rats was 93% type I and 7% type IIa, whereas the cumulative plantaris fiber type percentage for all rats was 7% type I, 24% type IIa and 69% type IIx. Representative soleus and plantaris micographs are presented in Figure 2C.

### Differences in plantaris and soleus fiber cross sectional area, myonuclear number, and markers of transcriptional capacity between age groups

**Figure 3:**
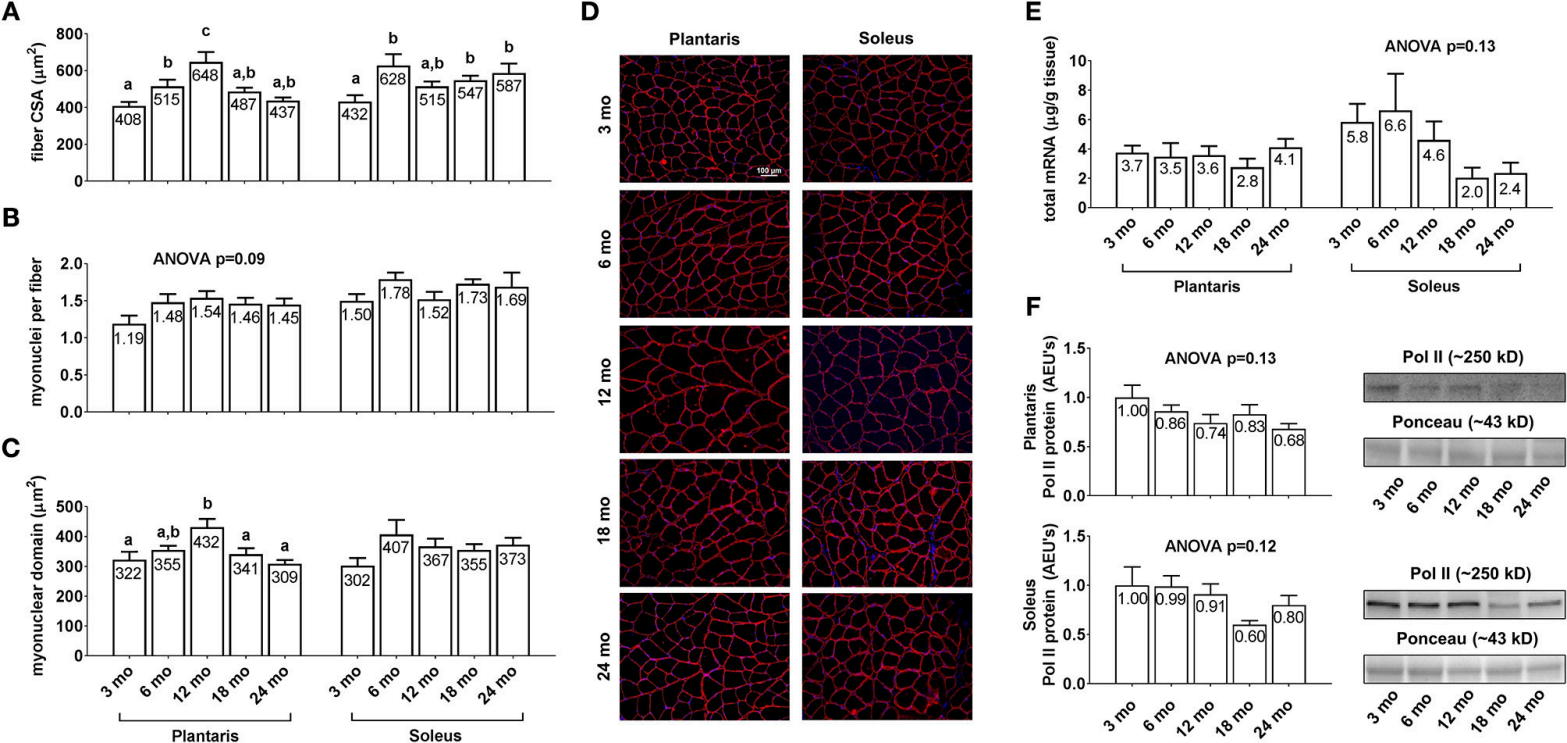
Effect of aging on muscle fiber CSA, myonuclear number, myonuclear domain size, and markers of transcriptional capacity. Age group differences in plantaris and soleus fiber CSA's **(A)**, plantaris and soleus myonuclear number per fiber **(B)**, plantaris and soleus myonuclear domain area **(C)**, plantaris and soleus total mRNA levels **(E)** and plantaris and soleus Poll II protein levels **(F)**. **(D)** depicts representative images of plantaris and soleus muscles from each age group using a 20x objective (scale bar = 100 μm). Data are presented as mean + SE and mean values for each dependent variable are presented within each bar. Different superscript letters indicate significant between-group differences indicated by one-way ANOVAs and protected LSD *post-hoc* tests (*p* < 0.05). AEU's, arbitrary expression units; mo, month.

Plantaris fiber CSA (Figure 3A, left inset) was 26% greater in 6 month vs. 3 month rats (*p* < 0.05), 26% greater in 12 month vs. 6 month rats (*p* < 0.05), 33% lower in 18 month vs. 12 month rats (*p* < 0.05), and not statistically different between 18 and 24 month rats. Soleus fiber CSA (Figure 3A, right inset) was 26–45% greater in the 6/18/24 month groups vs. 3 month rats (*p* < 0.05), and not statistically different between the 6/12/18/24 month groups. Plantaris and soleus myonuclear number per fiber (Figure 3B) were not statistically different between the any of the age groups. Plantaris myonuclear domain area (Figure 3C, left inset) was not statistically different between the 3/6/18/24 month groups, although it was 27–34% higher in 12 month rats vs. the 3/18/24 month groups. Soleus myonuclear domain area (Figure 3C, right inset), plantaris/soleus total RNA (Figure 3E), and plantaris/soleus Pol II protein levels (Figure 3F) were not statistically different between the any of the age groups. Representative soleus and plantaris micographs from each age group are presented in Figure 3D.

### Differences in plantaris and soleus markers of protein turnover and oxidative stress between age groups

**Figure 4:**
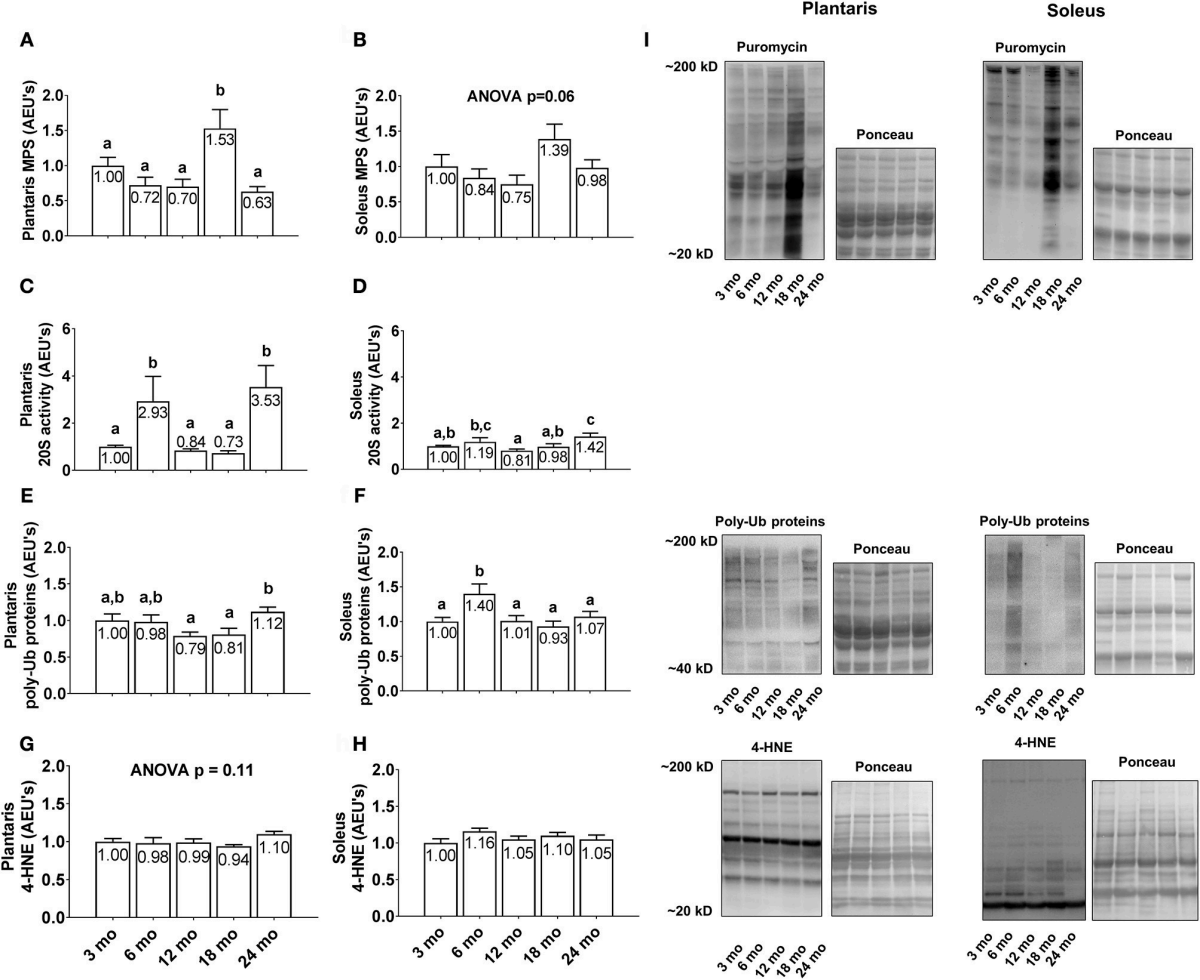
Effect of aging on plantaris and soleus markers of protein turnover and oxidative stress. Age group differences in plantaris and soleus muscle protein synthesis (MPS) levels **(A,B)**, proteasome activity levels **(C,D)**, poly-ubiquinated (poly-Ub) protein levels **(E,F)**, and 4-HNE levels **(G,H)**. **(I)** Depicts representative Western blot and Ponceau images of these markers from each age group. Data are presented as mean + SE and mean values for each dependent variable are presented within each bar. Different superscript letters indicate significant between-group differences indicated by one-way ANOVAs and protected LSD *post-hoc* tests (*p* < 0.05). AEU's, arbitrary expression units; mo, month.

Plantaris MPS levels were greatest in 18 month rats with values significantly higher than all other age groups (Figure 4A; *p* < 0.05), although there were no statistical differences in soleus MPS levels between age groups (Figure 4B; *p* < 0.05). Plantaris 20S proteasome activity was robustly up-regulated in the 6 and 24 month groups compared to all other age groups (*p* < 0.05; Figure 4C), whereas soleus 20S proteasome activity was significantly greater in 6 month vs. 12 month rats and 24 month vs. 3/12/18 month rats (*p* < 0.05; Figure 4D). Plantaris poly-Ub protein levels were not statistically different between the 3/6/12/18 month groups, although levels were ~40% greater in 24 month vs. 12 and 18 month rats (Figure 4E; *p* < 0.05). Soleus poly-Ub protein levels were not statistically different between the 3/12/18/24 month groups, although levels were ~40% greater in 6 month vs. 3/12/18/24 month rats (Figure 4F; *p* < 0.05). There were no differences between age groups regarding plantaris or soleus 4-HNE levels (Figures 4G,H), which is a well validated marker of lipid peroxidation (Zhong and Yin, 2015). Representative soleus and plantaris Western blot images from each age group are presented in Figure 4I.

### Differences in plantaris and soleus regulators of translation between age groups

**Figure 5:**
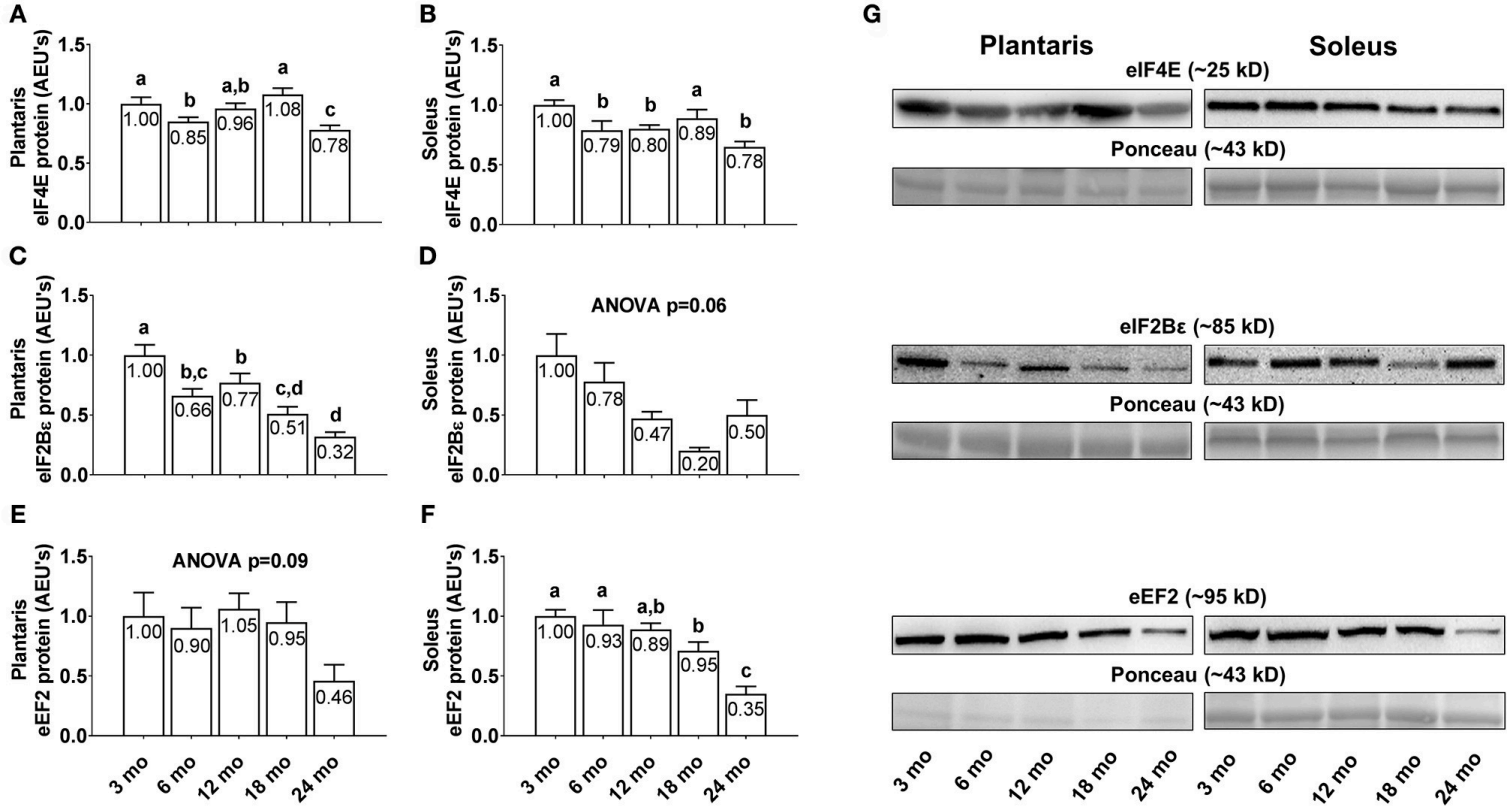
Effect of aging on select eukaryotic initiation and elongation factors in plantaris and soleus muscle. Age group differences in plantaris and soleus muscle eukaryotic initiation factor (eIF) 4E protein levels **(A,B)**, plantaris and soleus muscle eIF2Bε protein levels **(C,D)**, and plantaris and soleus muscle eukaryotic elongation factor (eEF) 2 protein levels **(E,F)**. **(G)** Depicts representative Western blot and Ponceau images of these markers from each age group. Data are presented as mean ± SE and mean values for each dependent variable are presented within each bar. Different superscript letters indicate significant between-group differences indicated by one-way ANOVAs and protected LSD *post-hoc* tests (*p* < 0.05). AEU's, arbitrary expression units; mo, month.

Plantaris eIF4E protein levels were 15% greater in 3 month vs. 6 month rats (Figure 5A; *p* < 0.05), 27% greater in 18 month vs. 6 month rats, and lower in 24 month rats compared to all other age groups (*p* < 0.05). Soleus eIF4E protein levels were significantly higher in 3 and 18 month rats compared to all other age groups (Figure 5B; *p* < 0.05), though not significantly different from each other. Plantaris eIF2Bε protein levels displayed a near-linear decrease across age groups, as they were significantly higher in 3 month rats compared to all other age groups (Figure 5C; *p* < 0.05), and lower in 24 month rats vs. the 3/6/12 month groups (*p* < 0.05). Soleus eIF2Bε protein levels were not statistically different between age groups (Figure 5D; ANOVA *p* = 0.06), albeit these levels also trended downward with aging. Plantaris eEF2 protein levels were not statistically different between age groups (Figure 5E; ANOVA *p* = 0.09), although these levels were >50% lower in 24 month rats compared to all other age groups. Soleus eEF2 protein levels were not statistically different between the 3/6/12 month groups, although they were significantly lower in 18 month rats vs. the 3/6 month groups and ~70% lower in the 24 month rats compared to all other age groups (Figure 5F; *p* < 0.05). Representative soleus and plantaris Western blot images from each age group are presented in Figure 5G.

### Differences in plantaris and soleus markers of ribosome biogenesis and ribosome density between age groups

**Figure 6:**
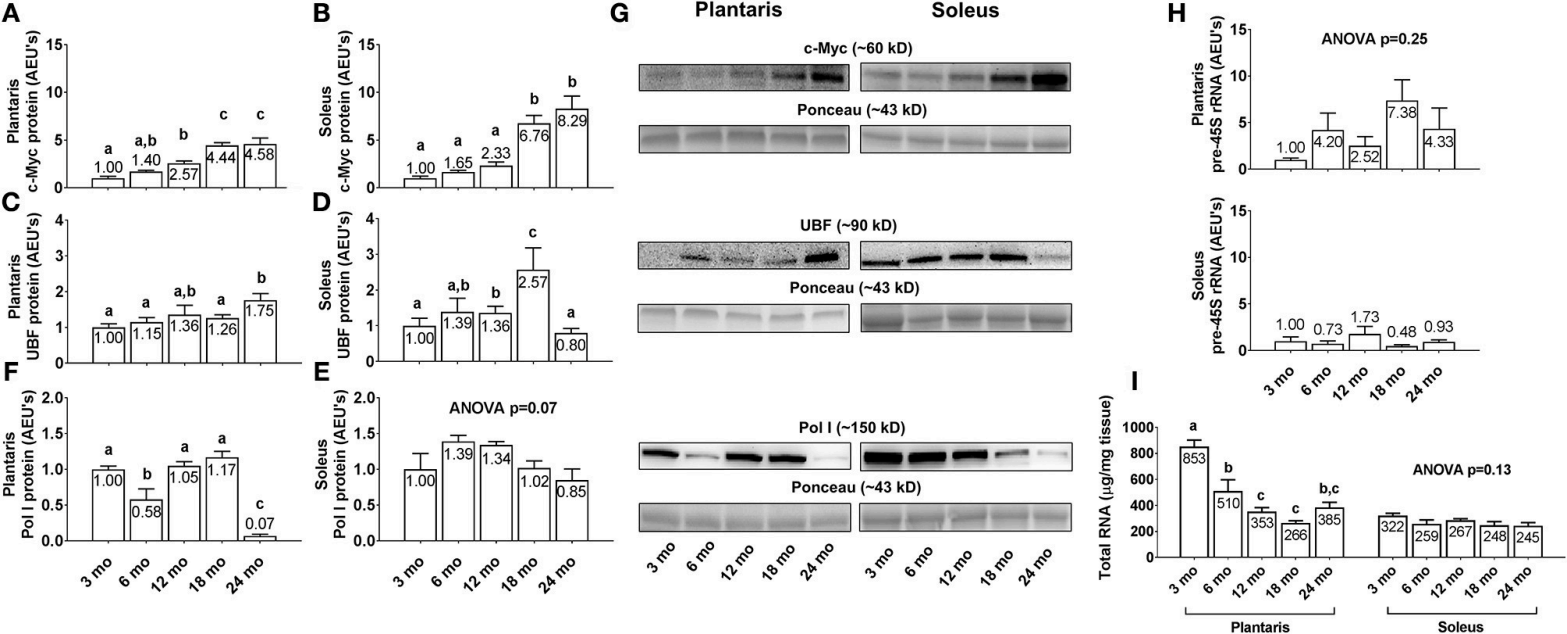
Effect of aging on select markers of ribosome biogenesis and ribosome density in plantaris and soleus muscle. Age group differences in plantaris and soleus muscle v-myc avian myelocytomatosis viral oncogene homolog (c-Myc) protein levels **(A,B)**, plantaris and soleus muscle upstream binding factor (UBF) protein levels **(C,D)**, plantaris and soleus muscle RNA polymerase (Pol) I protein levels **(E,F)**, plantaris and soleus pre-45S ribosomal RNA levels **(H)** and plantaris and soleus total RNA levels **(I)**. **(G)** Depicts representative Western blot and Ponceau images of c-Myc, UBF, and Pol I from each age group. Data are presented as mean ± SE and mean values for each dependent variable are presented within each bar. Different superscript letters indicate significant between-group differences indicated by one-way ANOVAs and protected LSD *post-hoc* tests (*p* < 0.05). AEU's, arbitrary expression units; mo, month.

Plantaris and soleus c-Myc protein levels (Figures 6A,B) increased linearly with aging. Specifically, c-Myc levels were highest in both muscles in the 24 month rats, and levels were 4.6-fold and 8.3-fold greater in the plantaris and soleus muscles of 24 month vs. 3 month rats, respectively (*p* < 0.05). Plantaris UBF protein levels were greater in 24 month vs. the 3/6/18 month groups (Figure 6C; *p* < 0.05), and soleus UBF protein levels were significantly higher in 18 month rats compared to all other age groups (Figure 6D; *p* < 0.05). Plantaris Pol I protein levels were lower in 6 month vs. the 3/12/18 month groups (Figure 6E; *p* < 0.05), were not statistically different between the 3/12/18 month groups, and were drastically lower (~87–94%) in the 24 month rats compared to all other groups (*p* < 0.05). Soleus Pol I protein levels (Figure 6F) and plantaris/soleus pre-45S rRNA levels (Figure 6H) were not statistically different between groups. Plantaris total RNA levels (Figure 6I, left inset) significantly higher in 3 month rats compared to all other age groups (*p* < 0.05), significantly higher in 6 month rats compared to the 12/18 month groups, and were not statistically different between the 12/18/24 month groups. Soleus total RNA levels (Figure 6I, right inset) were not statistically different between groups. Representative soleus and plantaris Western blot images from each age group are presented in Figure 6G.

### Select correlations examining potential contributors to age-related plantaris fiber atrophy in 12 month vs. 24 month rats

**Figure 7:**
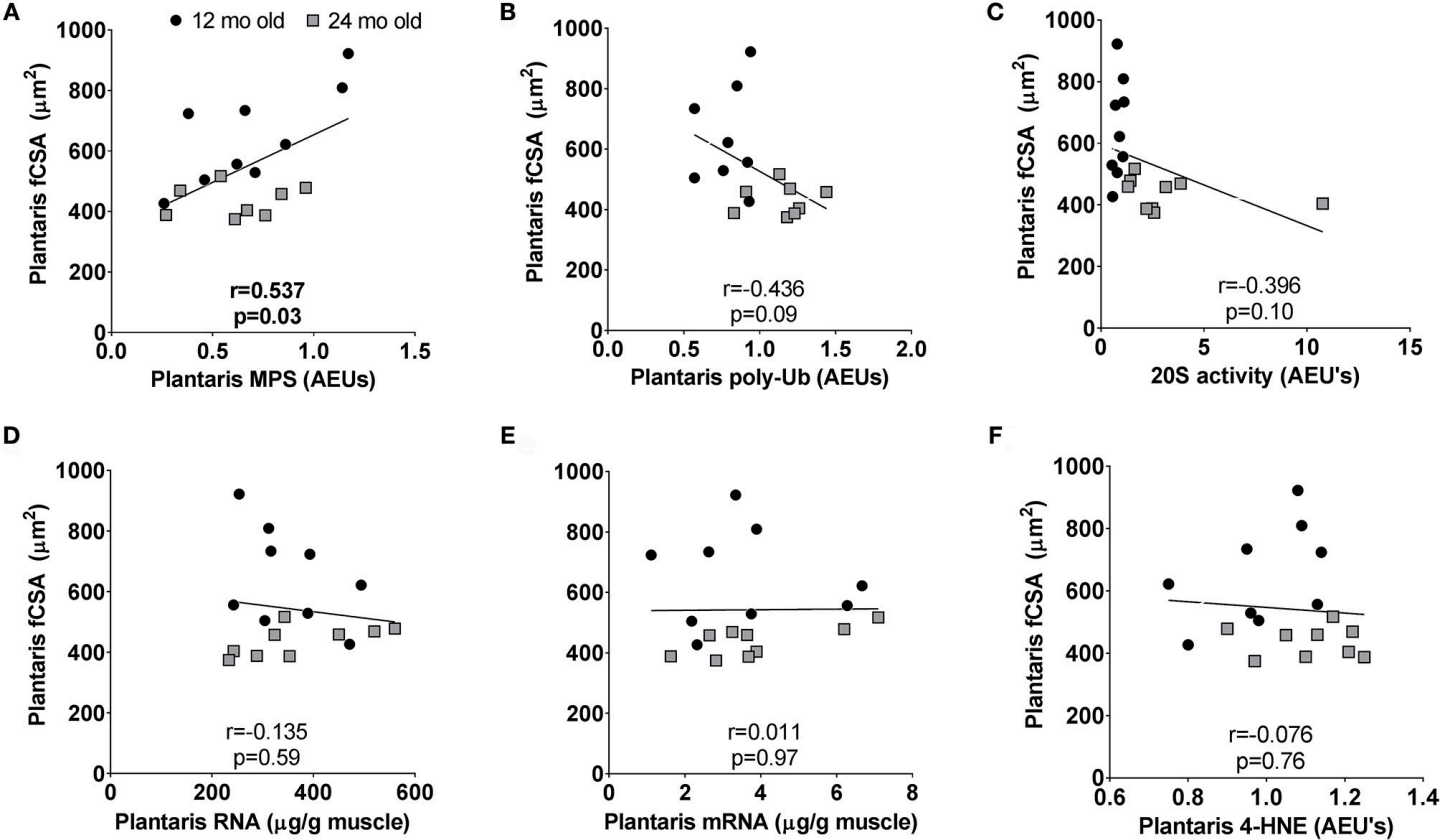
Select correlations examining potential contributors to age-related fast-twitch fiber atrophy in 12 month vs. 24 month rats. Correlations were performed between plantaris CSA values and MPS levels **(A)**, poly-ubiquinated protein levels **(B)**, 20S proteasome activity levels **(C)**, ribosome density (or total RNA) **(D)**, total mRNA **(E)**, and 4-hydroxynonenal (4-HNE) levels **(F)**. mo, month.

Plantaris fiber CSA values were highest in 12 month rats indicating this age was near peak lifetime values, whereas atrophy was evident when comparing plantaris fiber CSA values between 24 month vs. 12 month rats. Hence, we performed correlations between plantaris CSA values and select markers of transcriptional output (total mRNA), translational capacity (MPS levels and total RNA), and muscle proteolysis (proteasome activity and poly-Ub protein levels) in the 12 month vs. 24 month groups to determine how these variables are associated age-related muscle fiber CSA changes. These associations were not performed in the soleus muscle given that age-related fiber atrophy did not occur. There was a significant positive association between plantaris fiber CSA and MPS levels (*r* = 0.537, *p* = 0.03; Figure 7A), and a negative associations trended between fiber CSA and poly-Ub protein levels (*r* = −0.436, *p* = 0.09; Figure 7B) as well as 20S proteasome activity (*r* = −0.396, *p* = 0.10; Figure 7C). There were no associations with plantaris fiber CSA and ribosome density (total RNA) (Figure 7D), total mRNA (Figure 7E) or 4-HNE (Figure 7F).

## Discussion

Aging is associated with increased functional limitations due to a loss of lean muscle mass, strength, and power (Manini et al., 2013). While age-related muscle loss is due in part to decreases in MPS (i.e., ribosome function), other biological mechanisms which coincide with or contribute to muscle loss are of great interest to researchers that study aging. Herein, we observed age-related declines in plantaris fiber CSA (−33% from 12 to 24 months) and increases in soleus fiber CSA (+36% from 3 to 24 months). Our findings are in agreement with rodent (Holloszy et al., 1991) and human studies (Verdijk et al., 2007; Snijders et al., 2009; Nilwik et al., 2013) reporting that type II muscle fibers undergo age-related atrophy. Raw plantaris muscle weights exhibited peak values in 6/12/18 month rats and decrements were evident in 24 month rats, whereas relative (body mass-adjusted) plantaris masses decreased in an age-related fashion after peak values were observed in 6 month rats. It is notable, however, that relative plantaris muscle mass decreases across age groups can be largely influenced by increases in whole-body adiposity as rat age increases (unpublished observations). We also contend that fiber CSA assessment likely provides a more sensitive measurement to alterations in skeletal muscle size when compared to analyzing whole or relative muscle tissue weights. Thus, the highest raw plantaris masses and fiber CSA values observed in 12 month rats likely indicates a value reflective of peak muscle mass at this age group.

Select correlations in 12 month (peak plantaris CSA) and 24 month rats (the age of plantaris atrophy manifestation) reveal that higher plantaris MPS levels were significantly associated with greater CSA values, whereas CSA values were not significantly associated with total mRNA, ribosome density (total RNA), or 4-HNE levels. Negative associations approached significance whereby higher poly-Ub protein and 20S proteasome activity levels were associated lower plantaris fiber CSA values. Thus, these associations collectively indicate that age-related plantaris atrophy from 12 to 24 month rats are better explained through MPS decrements and potential increases in muscle proteolysis rather than alterations in transcriptional capacity, muscle ribosome content and/or oxidative stress. Contrary to this hypothesis, however, was the observation of higher MPS levels in 18 month vs. 12 month rats (+85%) as well as a sharp MPS decrement in 24 month vs. 18 month rats (−30%). While it is difficult to reconcile why plantaris MPS levels were greatest in 18 month rats despite the onset of fiber atrophy, these data are in partial agreement with Kimball et al. (2004) who reported that gastrocnemius total protein synthesis and myofibril protein synthesis rates were 2-fold higher in 24 month vs. 6 month male Sprague Dawley rats despite lower muscle weights in the 24 month group. The authors posited that the concomitant increase in mammalian target of rapamycin signaling complex 1 (mTORC1) proteins and select eukaryotic initiation factors was likely responsible for MPS increases in an attempt to mitigate muscle atrophy (Kimball et al., 2004). While our data is not in agreement in this regard given that plantaris eIF4E and eIF2Bε presented age-related decrements (not increases), we agree with the rationale of Kimball et al. in that the high MPS levels in 18 month rats may be some type of compensatory mechanism that is up-regulated by muscle to combat age-related muscle atrophy.

The near-linear decrease in plantaris ribosome density (i.e., total RNA) across age groups was another notable observation despite the fact that this variable was not associated with plantaris fiber CSA decrements from 12 to 24 months. While this observation contrasts prior literature reporting that skeletal muscle ribosome density is similar in younger vs. older humans (Roberts et al., 2010; Stec et al., 2015) or is greater in older rats (Haddad and Adams, 2006), this finding may be due to our examination of the plantaris muscle vs. the examination of *vastus lateralis* tissue in humans or gastrocnemius muscles in rats in the aforementioned studies; both which contain a heterogeneous mixture of type I and II fibers. Notably, soleus muscle total RNA levels did not differ between age groups which suggests that slow-twitch fiber ribosome density does not decrease with aging in Fisher rats. Thus, this finding supports the notion that sampling a relatively heterogeneous fiber-typed muscle (e.g., vastus lateralis or gastrocnemius) may mask potential decrements in fast-twitch fiber ribosome density.

Interestingly, plantaris c-Myc and UBF protein levels (i.e., transcriptional regulators of rRNA) paradoxically increased with aging, and (while not significant) pre-45S rRNA transcript levels were relatively greater in 18 month (738%) and 24 month rats (433%) compared to 3 month rats. Similar to the plantaris MPS data presented above, these data collectively suggest that transcriptional regulators of ribosome biogenesis in fast-twitch fibers, and perhaps the initial stages of ribosome biogenesis itself (i.e., rDNA transcription), may increase through a compensatory mechanism in an attempt to offset the sharp decline in ribosome density with aging. However, given that total RNA decrements persisted with aging, mechanisms related to rRNA processing or nuclear ribosome export may be impeded and/or ribosome turnover may be increased with aging in plantaris muscle. This hypothesis is not unfounded given that Kirby et al. (2015) reported increases in plantaris total RNA levels are drastically impaired in 25 month vs. 5 month mice in response to synergist ablation. We also observed a sharp decline in plantaris RNA Pol I protein levels from 18 to 24 months. RNA pol I functions as the enzyme which catalyzes rDNA transcription for ribosome biogenesis (Chaillou et al., 2014). Hence, this finding suggests that RNA Pol I-mediated ribosome biogenesis may be greatly impaired in the fast-twitch fibers of rats that exceed 24 months of age which, in turn, likely leads to a further decrease in ribosome number and MPS past 24 months in Fisher rats. Plantaris total mRNA, myonuclear number and RNA Pol II, the latter which is the enzyme that catalyzes mRNA transcription (Chaillou et al., 2014), were not altered across the studied age spectrum. Thus, while there are well-known changes in select mRNAs that coincide with and potentially contribute to muscle aging (i.e., inflammation-associated mRNAs, cell cycle-related mRNAs, and mRNAs associated with fibrosis) (Melov et al., 2007; Raue et al., 2012), our data suggest that global mRNA transcriptional capacity may not be associated with age-related type II fiber atrophy.

Regarding the age-related soleus muscle alterations observed herein, we report that fiber CSA values remained stable from 6 to 24 months. As mentioned previously, type I fiber atrophy is more resilient to aging, and researchers have hypothesized that this phenomenon is due to these fibers being continuously activated during weight-bearing activities relative to type II fibers which are only activated through high-intensity exercise or resistance training endeavors (Fielding et al., 2011). Hence, it is plausible that this aging effect led to relatively lower plantaris MPS levels and the age-related decline in plantaris ribosome density in 24 month rats. However, it is also notable that soleus MPS levels and ribosome density did not increase across age groups in a compensatory fashion during plantaris atrophy which suggests that the maintenance in soleus fiber size across age group were not driven through ribosome density or function increases. In terms of other age-associated mechanisms observed in the soleus, it is notable that poly-Ub levels did significantly increase (+40%) from 3 to 6 months, albeit levels remained low in the 12/18/24 month groups, and 20S activity levels were modestly higher in the 24 month rats compared to all other age groups. Soleus UBF protein levels were also significantly greater in 18 month vs. 24 month rats, and eEF2 protein levels also decreased across the age spectrum in a linear fashion. However, again, all of these aforementioned phenomena were seemingly unrelated to soleus fiber size given that soleus CSA values remained stable from 6 to 24 month rats. Interestingly, soleus mRNA levels trended downward with aging; specifically, these values decreased ~60% from peak values in 24 month vs. 6 month rats although these were statistically non-significant decreases. This phenomenon was not seemingly related to a reduction in myonuclear number and/or a decreased expression of RNA pol II protein levels given that there were no significant age-associated decreases in these variables. Others have reported that aging reduces total mRNA levels in the brain cortex (Zs-Nagy and Semsei, 1984) and liver (De Cecco et al., 2013), and the authors from these studies hypothesized that age-related tissue mRNA decrements may be due to a potassium-induced condensation of chromatin and/or an increased expression of histone proteins which leads to chromatin packaging. However, while these mechanisms warrant further investigation, we speculate that age-related decrements in soleus transcriptional capacity is not a likely culprit involved in slow-twitch fiber atrophy given that soleus CSA did not decline with aging in our current model.

What should finally be noted is the age-related fiber type phenomena observed in the current study. Fiber typing was initially performed in order to validate our assumption that the assayed plantaris muscles were composed of mainly fast twitch (or type II) fibers, whereas soleus muscles were composed of slow twitch (or type I) fibers. However, interesting observations were noted including the following: (a) soleus fiber type was not statistically different between age groups, (b) while the ANOVA for plantaris type I fiber percentage approached significance, there was a clear numerical difference between age groups whereby 24 month rats possessed 13% type I fibers and younger age groups possessed 2–9% type I fibers, and (c) 24 month rats possessed the lowest number of type IIa fibers compared to all other age groups. Hence, these data collectively suggest a potential fiber type shift in the plantaris muscle whereby older rats may lose type IIa fibers thereby increasing the proportion of type I fibers, and this phenomena has been reported in past human (Miljkovic et al., 2015) and rodent literature (Putman et al., 2001). While studying the potential mechanisms of this age-related fiber type shift was beyond the realm of the current investigation, future research should continue to examine the mechanisms that drive this phenotypic change.

### Experimental considerations

This study is not without limitations. One limitation is that our report observes molecular alterations in the plantaris and soleus muscles over the lifespan of rats without the direct measurement of functional alterations that may be occurring in these muscles (i.e., peak tetanic tension and/or specific tension). However, forelimb strength as well as rotarod performance linearly decreases in an age-related fashion in these rats and posit that force-generating abilities of the assayed muscles likely also decline in a similar fashion. Another limitation that should be noted that only male rats were studied herein. In this regard, replicating this study design in female rats would yield invaluable insight as to how aging in females affects these same molecular markers. Aside from the aforementioned limitations, one experimental consideration should be also noted; specifically, while we studied rats up to 24 months of age, it should be noted that other studies have examined the Fisher 344xBrown Norway strain up to 39 months of age and have reported muscle mass and fiber loss occur at 30–33 months (Lushaj et al., 2008). We report, however, that plantaris fiber atrophy occurs by 18 months in Fisher rats and that robust decrements in select regulators of transcriptional and translational capacity in skeletal muscle also occur by 24 months in these rats; these phenomena being potentially related to the fact that the average life expectancy for Fisher rats has been reported to be 25 months whereas Fisher 344xBrown Norway has been reported to be 36–37 months (Lipman et al., 1996).

## Conclusions

We contend that our data warrant future experiments which continue to elucidate how aging affects fast-twitch skeletal muscle physiology. Our report that plantaris fiber CSA, MPS levels and ribosome content were downregulated while 20S proteasome activity levels were elevated in 24 month rats suggests that multiple signaling pathways are disrupted in Male Fisher rats following 18 months of age. Indeed, other researchers have posited that the age-related culmination of skeletal muscle oxidative stress and subsequent mitochondrial defects is a primary mechanism of skeletal muscle aging (Zuo and Pannell, 2015). Moreover, age-related increases in oxidative stress may decrease ribosome function (discussed more below) while also increasing proteolysis (Costa et al., 2007) suggesting that increased oxidative stress may be responsible for the phenomena we observed in the current study. Our preliminary 4-HNE data seemingly suggests that global oxidative stress in the plantaris muscles of older rats is not increased. However, an intriguing future research aim would be to examine how skeletal muscle aging specifically affect oxidative stress of ribosomal components. In this regard, data performed in yeast suggests that experimentally-induced oxidative stress increased ribosomal translation errors (i.e., stop codon read-through and frameshift events) (Gerashchenko et al., 2012), and others have also reported that charged transfer RNA (tRNA) synthesis is impaired *in vitro* during experimentally-induced oxidative stress which, again, could functionally impair ribosome function and/or lead to the faulty synthesis of skeletal muscle proteins (Netzer et al., 2009). Hence, future *in vivo* research is needed in order to clarify if ribosomes in older skeletal muscle present impairments in MPS via increased oxidative stress to ribosomal proteins, rRNA and/or tRNA. In conclusion, these data provide meaningful details regarding how aging affects select markers of ribosome biogenesis, ribosome function/capacity, transcriptional capacity, and markers of proteolysis and oxidative stress in slow- and fast-twitch rodent muscle.

## Author contributions

CM oversaw most experiments, and CM and MR primarily drafted the manuscript. JW and RL provided a contract to MR to perform the experiments, and all three authors were critically involved study design. All other authors assisted with data analysis and editing, and all authors approved the final version of manuscript.

### Conflict of interest statement

The authors declare that the research was conducted in the absence of any commercial or financial relationships that could be construed as a potential conflict of interest.

## References

[B1] Aoi W. Sakuma K. (2011). Oxidative stress and skeletal muscle dysfunction with aging. Curr. Aging Sci. 4, 101–109. 10.2174/1874609811104020101 21235498

[B2] Balagopal P. Rooyackers O. E. Adey D. B. Ades P. A. Nair K. S. (1997). Effects of aging on *in vivo* synthesis of skeletal muscle myosin heavy-chain and sarcoplasmic protein in humans. Am. J. Physiol. 273, E790–E800. 9357810 10.1152/ajpendo.1997.273.4.E790

[B3] Bennett B. T. Mohamed J. S. Alway S. E. (2013). Effects of resveratrol on the recovery of muscle mass following disuse in the plantaris muscle of aged rats. PLoS ONE 8:e83518. 10.1371/journal.pone.0083518 24349525 PMC3861503

[B4] Chaillou T. (2017). Impaired ribosome biogenesis could contribute to anabolic resistance to strength exercise in the elderly. J. Physiol. (Lond). 595, 1447–1448. 10.1113/JP273773 28105708 PMC5330918

[B5] Chaillou T. Kirby T. J. McCarthy J. J. (2014). Ribosome biogenesis: emerging evidence for a central role in the regulation of skeletal muscle mass. J. Cell. Physiol. 229, 1584–1594. 10.1002/jcp.24604 24604615 PMC4868551

[B6] Costa V. Quintanilha A. Moradas-Ferreira P. (2007). Protein oxidation, repair mechanisms and proteolysis in Saccharomyces cerevisiae. IUBMB Life 59, 293–298. 10.1080/15216540701225958 17505968

[B7] Cuthbertson D. Smith K. Babraj J. Leese G. Waddell T. Atherton P.. (2005). Anabolic signaling deficits underlie amino acid resistance of wasting, aging muscle. FASEB J. 19, 422–424. 10.1096/fj.04-2640fje 15596483

[B8] Deacon R. M. (2013). Measuring the strength of mice. J. Vis. Exp. 76:2610 10.3791/2610 PMC3725666 23770643

[B9] De Cecco M. Criscione S. W. Peterson A. L. Neretti N. Sedivy J. M. Kreiling J. A. (2013). Transposable elements become active and mobile in the genomes of aging mammalian somatic tissues. Aging (Albany. NY). 5, 867–883. 10.18632/aging.100621 24323947 PMC3883704

[B10] Doherty T. J. (2003). Invited review: aging and sarcopenia. J. Appl. Physiol. (1985) 95, 1717–1727. 10.1152/japplphysiol.00347.2003 12970377

[B11] Fielding R. A. Vellas B. Evans W. J. Bhasin S. Morley J. E. Newman A. B.. (2011). Sarcopenia: an undiagnosed condition in older adults. Current consensus definition: prevalence, etiology, and consequences. International working group on sarcopenia. J. Am. Med. Dir. Assoc. 12, 249–256. 10.1016/j.jamda.2011.01.003 21527165 PMC3377163

[B12] Gerashchenko M. V. Lobanov A. V. Gladyshev V. N. (2012). Genome-wide ribosome profiling reveals complex translational regulation in response to oxidative stress. Proc. Natl. Acad. Sci. U.S.A. 109, 17394–17399. 10.1073/pnas.1120799109 23045643 PMC3491468

[B13] Goodman C. A. Hornberger T. A. (2013). Measuring protein synthesis with SUnSET: a valid alternative to traditional techniques? Exerc. Sport Sci. Rev. 41, 107–115. 10.1097/JES.0b013e3182798a95 23089927 PMC3951011

[B14] Goodpaster B. H. Park S. W. Harris T. B. Kritchevsky S. B. Nevitt M. Schwartz A. V.. (2006). The loss of skeletal muscle strength, mass, and quality in older adults: the health, aging and body composition study. J. Gerontol. A Biol. Sci. Med. Sci. 61, 1059–1064. 10.1093/gerona/61.10.1059 17077199

[B15] Haddad F. Adams G. R. (2006). Aging-sensitive cellular and molecular mechanisms associated with skeletal muscle hypertrophy. J. Appl. Physiol. (1985) 100, 1188–1203. 10.1152/japplphysiol.01227.2005 16373446

[B16] Hamm R. J. Pike B. R. O'Dell D. M. Lyeth B. G. Jenkins L. W. (1994). The rotarod test: an evaluation of its effectiveness in assessing motor deficits following traumatic brain injury. J. Neurotrauma 11, 187–196. 10.1089/neu.1994.11.187 7932797

[B17] Holloszy J. O. Chen M. Cartee G. D. Young J. C. (1991). Skeletal muscle atrophy in old rats: differential changes in the three fiber types. Mech. Ageing Dev. 60, 199–213. 10.1016/0047-6374(91)90131-I 1745075

[B18] Hyatt H. W. Toedebusch R. G. Ruegsegger G. Mobley C. B. Fox C. D. McGinnis G. R.. (2015). Comparative adaptations in oxidative and glycolytic muscle fibers in a low voluntary wheel running rat model performing three levels of physical activity. Physiol. Rep. 3:e12619. 10.14814/phy2.12619 26603455 PMC4673647

[B19] Kadi F. Charifi N. Denis C. Lexell J. (2004). Satellite cells and myonuclei in young and elderly women and men. Muscle Nerve 29, 120–127. 10.1002/mus.10510 14694507

[B20] Kerksick C. M. Roberts M. D. Dalbo V. J. Sunderland K. L. (2016). Intramuscular phosphagen status and the relationship to muscle performance across the age spectrum. Eur. J. Appl. Physiol. 116, 115–127. 10.1007/s00421-015-3246-1 26307531

[B21] Kimball S. R. O'Malley J. P. Anthony J. C. Crozier S. J. Jefferson L. S. (2004). Assessment of biomarkers of protein anabolism in skeletal muscle during the life span of the rat: sarcopenia despite elevated protein synthesis. Am. J. Physiol. Endocrinol. Metab. 287, E772–E780. 10.1152/ajpendo.00535.2003 15187001

[B22] Kimball S. R. Vary T. C. Jefferson L. S. (1992). Age-dependent decrease in the amount of eukaryotic initiation factor 2 in various rat tissues. Biochem. J. 286(Pt 1), 263–268. 10.1042/bj2860263 1381583 PMC1133049

[B23] Kirby T. J. Lee J. D. England J. H. Chaillou T. Esser K. A. McCarthy J. J. (2015). Blunted hypertrophic response in aged skeletal muscle is associated with decreased ribosome biogenesis. J. Appl. Physiol. (1985) 119, 321–327. 10.1152/japplphysiol.00296.2015 26048973 PMC4538281

[B24] Kragstrup T. W. Kjaer M. Mackey A. L. (2011). Structural, biochemical, cellular, and functional changes in skeletal muscle extracellular matrix with aging. Scand. J. Med. Sci. Sports 21, 749–757. 10.1111/j.1600-0838.2011.01377.x 22092924

[B25] Kumar A. Accorsi A. Rhee Y. Girgenrath M. (2015). Do's and don'ts in the preparation of muscle cryosections for histological analysis. J. Vis. Exp. 15:e52793 10.3791/52793 PMC4542757 26066009

[B26] Lexell J. Taylor C. C. Sjostrom M. (1988). What is the cause of the ageing atrophy? Total number, size and proportion of different fiber types studied in whole vastus lateralis muscle from 15- to 83-year-old men. J. Neurol. Sci. 84, 275–294. 10.1016/0022-510X(88)90132-3 3379447

[B27] Lipman R. D. Chrisp C. E. Hazzard D. G. Bronson R. T. (1996). Pathologic characterization of brown Norway, brown Norway x Fischer 344, and Fischer 344 x brown Norway rats with relation to age. J. Gerontol. A Biol. Sci. Med. Sci. 51, B54–59. 10.1093/gerona/51A.1.B54 8548501 PMC7110307

[B28] Lushaj E. B. Johnson J. K. McKenzie D. Aiken J. M. (2008). Sarcopenia accelerates at advanced ages in Fisher 344xBrown Norway rats. J. Gerontol. A Biol. Sci. Med. Sci. 63, 921–927. 10.1093/gerona/63.9.921 18840796 PMC2902273

[B29] Manini T. M. Hong S. L. Clark B. C. (2013). Aging and muscle: a neuron's perspective. Curr. Opin. Clin. Nutr. Metab. Care 16, 21–26. 10.1097/MCO.0b013e32835b5880 23222705 PMC3868452

[B30] McClung J. M. Kavazis A. N. Deruisseau K. C. Falk D. J. Deering M. A. Lee Y.. (2007). Caspase-3 regulation of diaphragm myonuclear domain during mechanical ventilation-induced atrophy. Am. J. Respir. Crit. Care Med. 175, 150–159. 10.1164/rccm.200601-142OC 17082496 PMC1899279

[B31] Melov S. Tarnopolsky M. A. Beckman K. Felkey K. Hubbard A. (2007). Resistance exercise reverses aging in human skeletal muscle. PLoS ONE 2:e465. 10.1371/journal.pone.0000465 17520024 PMC1866181

[B32] Miljkovic N. Lim J. Y. Miljkovic I. Frontera W. R. (2015). Aging of skeletal muscle fibers. Ann. Rehabil. Med. 39, 155–162. 10.5535/arm.2015.39.2.155 25932410 PMC4414960

[B33] Mobley C. B. Fox C. D. Thompson R. M. Healy J. C. Santucci V. Kephart W. C. (2016a). Comparative effects of whey protein vs. L-leucine on skeletal muscle protein synthesis and markers of ribosome biogenesis following resistance exercise. Amino Acids 48, 733–750. 10.1007/s00726-015-2121-z 26507545

[B34] Mobley C. B. Mumford P. W. Kephart W. C. Conover C. F. Beggs L. A. Balaez A.. (2016b). Effects of testosterone treatment on markers of skeletal muscle ribosome biogenesis. Andrologia 48, 967–977. 10.1111/and.12539 26781353

[B35] Murgia M. Toniolo L. Nagaraj N. Ciciliot S. Vindigni V. Schiaffino S.. (2017). Single muscle fiber proteomics reveals fiber-type-specific features of human muscle aging. Cell Rep. 19, 2396–2409. 10.1016/j.celrep.2017.05.054 28614723

[B36] Nader G. A. McLoughlin T. J. Esser K. A. (2005). mTOR function in skeletal muscle hypertrophy: increased ribosomal RNA via cell cycle regulators. Am. J. Physiol. Cell Physiol. 289, C1457–C1465. 10.1152/ajpcell.00165.2005 16079186

[B37] Nair K. S. (2005). Aging muscle. Am. J. Clin. Nutr. 81, 953–963. 15883415 10.1093/ajcn/81.5.953

[B38] Netzer N. Goodenbour J. M. David A. Dittmar K. A. Jones R. B. Schneider J. R.. (2009). Innate immune and chemically triggered oxidative stress modifies translational fidelity. Nature 462, 522–526. 10.1038/nature08576 19940929 PMC2785853

[B39] Nilwik R. Snijders T. Leenders M. Groen B. B. Van Kranenburg J. Verdijk L. B.. (2013). The decline in skeletal muscle mass with aging is mainly attributed to a reduction in type II muscle fiber size. Exp. Gerontol. 48, 492–498. 10.1016/j.exger.2013.02.012 23425621

[B40] Piasecki M. Ireland A. Coulson J. Stashuk D. W. Hamilton-Wright A. Swiecicka A. (2016). Motor unit number estimates and neuromuscular transmission in the tibialis anterior of master athletes: evidence that athletic older people are not spared from age-related motor unit remodeling. Physiol. Rep. 4:e12987 10.14814/phy2.12987 27694526 PMC5064139

[B41] Putman C. T. Sultan K. R. Wassmer T. Bamford J. A. Skorjanc D. Pette D. (2001). Fiber-type transitions and satellite cell activation in low-frequency-stimulated muscles of young and aging rats. J. Gerontol. A Biol. Sci. Med. Sci. 56, B510–B519. 10.1093/gerona/56.12.B510 11723143

[B42] Raue U. Trappe T. A. Estrem S. T. Qian H. R. Helvering L. M. Smith R. C.. (2012). Transcriptome signature of resistance exercise adaptations: mixed muscle and fiber type specific profiles in young and old adults. J. Appl. Physiol. (1985) 112, 1625–1636. 10.1152/japplphysiol.00435.2011 22302958 PMC3365403

[B43] Roberts M. D. Kerksick C. M. Dalbo V. J. Hassell S. E. Tucker P. S. Brown R. (2010). Molecular attributes of human skeletal muscle at rest and after unaccustomed exercise: an age comparison. J. Strength Cond. Res. 24, 1161–1168. 10.1519/JSC.0b013e3181da786f 20440120

[B44] Schaap L. A. Pluijm S. M. Deeg D. J. Visser M. (2006). Inflammatory markers and loss of muscle mass (sarcopenia) and strength. Am. J. Med. 119, 526 e529–e517. 10.1016/j.amjmed.2005.10.049 16750969

[B45] Snijders T. Verdijk L. B. Van Loon L. J. (2009). The impact of sarcopenia and exercise training on skeletal muscle satellite cells. Ageing Res. Rev. 8, 328–338. 10.1016/j.arr.2009.05.003 19464390

[B46] Stec M. J. Mayhew D. L. Bamman M. M. (2015). The effects of age and resistance loading on skeletal muscle ribosome biogenesis. J. Appl. Physiol. (1985) 119, 851–857. 10.1152/japplphysiol.00489.2015 26294750 PMC4747892

[B47] Verdijk L. B. Koopman R. Schaart G. Meijer K. Savelberg H. H. Van Loon L. J. (2007). Satellite cell content is specifically reduced in type II skeletal muscle fibers in the elderly. Am. J. Physiol. Endocrinol. Metab. 292, E151–E157. 10.1152/ajpendo.00278.2006 16926381

[B48] Wen Y. Alimov A. P. McCarthy J. J. (2016). Ribosome biogenesis is necessary for skeletal muscle hypertrophy. Exerc. Sport Sci. Rev. 44, 110–115. 10.1249/JES.0000000000000082 27135313 PMC4911282

[B49] Zhong H. Yin H. (2015). Role of lipid peroxidation derived 4-hydroxynonenal (4-HNE) in cancer: focusing on mitochondria. Redox Biol. 4, 193–199. 10.1016/j.redox.2014.12.011 25598486 PMC4803793

[B50] Zs-Nagy I. Semsei I. (1984). Centrophenoxine increases the rates of total and mRNA synthesis in the brain cortex of old rats: an explanation of its action in terms of the membrane hypothesis of aging. Exp. Gerontol. 19, 171–178. 10.1016/0531-5565(84)90035-4 6207041

[B51] Zuo L. Pannell B. K. (2015). Redox characterization of functioning skeletal muscle. Front. Physiol. 6:338. 10.3389/fphys.2015.00338 26635624 PMC4649055

